# Beyond total treatment effects in RCTs: why we need to measure outcomes at baseline when investigating mediation

**DOI:** 10.1186/1745-6215-16-S2-O42

**Published:** 2015-11-16

**Authors:** Sabine Landau, Richard Emsley, Graham Dunn

**Affiliations:** 1 King's College London, London, UK; 2 University of Manchester, Manchester, UK

## Background

Randomisation in RCTs aims to avoid confounding bias when estimating the average treatment effect (ATE). For continuous outcomes measured post-treatment as well as before randomisation (baseline), analyses based on (i) post-treatment outcome alone, (ii) change scores over the treatment phase and (iii) conditioning on baseline values (ANCOVA), provide unbiased estimators of ATE with ANCOVA known to be most precise. The decision to include baseline values in the analysis is based on precision arguments.

## Methods

Investigators increasingly carry out explanatory analyses to partition total treatment effects into components that are mediated by an intermediate continuous outcome and a non-mediated part. Traditional mediation analysis might be performed based on (i) post-treatment values of the intermediate and clinical outcomes alone, (ii) respective change scores or (iii) conditioning mediator and clinical outcome models on their baseline values. Using Monte-Carlo simulation we investigated the performance of these competing estimators.

## Results

Approach (i) will lead to bias in estimates of causal mediation effects when a baseline variable affects both post-treatment variables, and when baseline values of either variable have themselves common causes. The change score approach (ii) can provide unbiased estimates under these confounding scenarios only under additional independence assumptions regarding baseline levels and change scores, while the conditioning approach (iii) is unbiased.

## Conclusions

Trialists envisaging mediation analyses should measure baseline values of continuous mediators and clinical outcomes. The conditioning approach is recommended to avoid bias in the presence of baseline confounding.

